# Reelin Functions, Mechanisms of Action and Signaling Pathways During Brain Development and Maturation

**DOI:** 10.3390/biom10060964

**Published:** 2020-06-26

**Authors:** Yves Jossin

**Affiliations:** Laboratory of Mammalian Development & Cell Biology, Institute of Neuroscience, Université Catholique de Louvain, 1200 Brussels, Belgium; yves.jossin@uclouvain.be

**Keywords:** embryonic development, postnatal maturation, cerebral cortex, neuron, migration, dendrites, synapse, Reelin, proteolytic processing, cellular pathways, signal transduction, neurodevelopmental disorders

## Abstract

During embryonic development and adulthood, Reelin exerts several important functions in the brain including the regulation of neuronal migration, dendritic growth and branching, dendritic spine formation, synaptogenesis and synaptic plasticity. As a consequence, the Reelin signaling pathway has been associated with several human brain disorders such as lissencephaly, autism, schizophrenia, bipolar disorder, depression, mental retardation, Alzheimer’s disease and epilepsy. Several elements of the signaling pathway are known. Core components, such as the Reelin receptors very low-density lipoprotein receptor (VLDLR) and Apolipoprotein E receptor 2 (ApoER2), Src family kinases Src and Fyn, and the intracellular adaptor Disabled-1 (Dab1), are common to most but not all Reelin functions. Other downstream effectors are, on the other hand, more specific to defined tasks. Reelin is a large extracellular protein, and some aspects of the signal are regulated by its processing into smaller fragments. Rather than being inhibitory, the processing at two major sites seems to be fulfilling important physiological functions. In this review, I describe the various cellular events regulated by Reelin and attempt to explain the current knowledge on the mechanisms of action. After discussing the shared and distinct elements of the Reelin signaling pathway involved in neuronal migration, dendritic growth, spine development and synaptic plasticity, I briefly outline the data revealing the importance of Reelin in human brain disorders.

## 1. Introduction

The reeler mouse was first described in 1951 as a spontaneous autosomal recessive mutation [1]. The name originates from the reeling gait observed in these mice along with tremor, ataxia and hypotonia. The cytoarchitectonic phenotype was described later and found similar in different mouse genetic backgrounds [2,3]. The most overt and maybe the most studied reeler phenotype is the abnormal layering of neurons in the brain. Reeler mutation causes neuronal ectopia in laminated brain structures such as cerebral and cerebellar cortices and the hippocampus, while more subtle anomalies are found in other parts of the brain including the olfactory bulb, spinal cord, thalamus, tectum, retina and amygdala [4,5,6,7,8]. In addition to its function in neuronal migration, Reelin regulates dendritic growth, dendritic spine development, and synapse formation and plasticity. Finally, Reelin is also expressed in nonneuronal tissues and evidence suggests the involvement of the canonical and noncanonical Reelin signaling pathways in the development of lymphatic vessels, mammary glands, submandibular glands, the small intestine, cartilage and bone. In the adult, Reelin has been suggested to have an effect on the immune system, liver fibrosis and multiple cancers [9].

Here, I will mostly focus on the functions of Reelin on excitatory neurons in the cerebral cortex as it is the most studied brain region when it comes to the Reelin pathway. This review addresses the current understanding of the structure, signaling pathway and mechanisms of action of Reelin and its potential link with human neurological disorders. Despite many advances in our knowledge of the pathway, several cellular and molecular aspects of the multiple functions of Reelin during embryonic development and in the postnatal and adult brain are still being elucidated. I will highlight the biological roles of Reelin signaling during neuronal migration, dendrite development and synapse formation. I will then propose hypotheses about the mechanisms of action and highlight differences in the signaling proteins involved. Finally, I will briefly outline the data revealing the importance of Reelin in human brain disorders.

## 2. Neuronal Migration Phenotype of the Reeler Mutant Mouse

### 2.1. Cell Mispositioning in the Neocortex

The early steps of cortical development occur normally in the reeler mouse. The germinal layer of the future telencephalon is a pseudostratified neuroepithelium primarily containing neural stem cells that proliferate symmetrically to increase their number and to expand the tissue laterally until embryonic day 11.5 (E11.5) in the mouse and gestation weeks 5–6 (GW 5–6) in humans [10,11]. Neocortical development and neurogenesis are initiated by the formation of the preplate (PP), also named the primordial plexiform layer, between the pial surface and the germinal layer, also called the ventricular zone (VZ). This phase occurs during GW 7 in humans and E12.5 in mice [12,13,14]. The PP is composed of pioneer neurons originating from inside and outside the cerebral cortex [15,16,17,18]. The PP is then invaded by glutamatergic excitatory projection (pyramidal) neurons to form the cortical plate (CP). They constitute the majority of neurons in the cerebral cortex and are produced at the VZ [19,20]. The first wave of future CP neurons migrates into and splits the PP to create an outer marginal zone (MZ), mostly populated by Cajal–Retzius cells, and an inner, transient subplate. The PP splitting occurs from GW 7–8 in humans and E13.5 in mice. In the reeler cerebral cortex, the PP develops normally, but neurons of the future CP cannot invade this structure that is rather displaced outward and becomes what is called the “superplate” [21,22].

During this early phase of cortical neurogenesis, the cerebral wall is thin. A short and straightforward movement is sufficient for newborn neurons to displace their cell body away from the proliferative zone. These early born neurons inherit a basal process which reaches the basal lamina from the division of the mother cell [23,24]. The nucleus and other organelles translocate within the elongated cytoplasm, while the neuron disassembles its junctions with surrounding cells and detaches from the apical surface [23,25,26,27]. This short movement is called “somal translocation”. In the reeler and wild-type mice, the first neurons that invade the PP (future layer VI neurons) are equivalently positioned beneath the pia during the period of PP splitting and initial CP formation [28]. However, mutant neurons remain intermingled with PP cells and show misoriented dendrites, while a recognizable nascent CP has formed in the wild-type cortex with dendrites of future layer VI neurons correctly oriented towards the pial surface. The next waves of neurons will continue to pile up beneath the superplate in the reeler mouse, while wild-type neurons widen the CP between the MZ and the subplate.

As the neocortex develops, the cerebral wall increases in thickness. The distances travelled by projection neurons reach several hundred microns in the mouse and range from 1 to 4 mm in humans [29]. The somal translocation mode of migration is gradually replaced by a radial migration subdivided into four steps: a short bipolar migration during which postmitotic cells move away from the VZ, followed by a multipolar migration. Cells then transit back to the bipolar locomotion and finish their journey with a terminal somal translocation [25,30,31,32]. During the locomotion mode of migration, neurons use the long radial glia fibers, which span the entire depth of the cerebral wall, as a substrate for their migration. On the other hand, like the somal translocation of early born neurons, the multipolar migration and terminal somal translocation of late born neurons are glia-independent [25,33].

Projection neurons migrate in successive waves to occupy progressively more superficial positions, migrating past the neurons already installed. This results in the development of an inside-out layering to form the 6-layered cortical structure with younger neurons in the outermost field of the cerebral wall and older neurons more inside [34,35,36]. The peak of neuronal migration in the mouse takes place from E12 to E16, while in humans, it occurs from GW 12 to 20 [37]. In the reeler neocortex, neurons are generated in normal numbers and at the normal time [38] but are not well organized into layers in an inside-out fashion. This was initially described as an inversion of the stereotypical layering [21]. However, even though the earliest neurons shift from a deep laminar position to form a superficially located superplate in the reeler brains, the later born neurons exhibit a broader and irregular distribution, which is far more than just an inversion of laminar fate [8,39].

### 2.2. Mechanism of Action of Reelin During Neocortical Neuron Migration

Several models have been proposed to explain the effect of Reelin on radially migrating neurons in the cerebral cortex. Reelin, which is secreted in the MZ by Cajal–Retzius cells [40], might serve as an attractant [41], enabling neurons to move beyond their predecessors that are already installed in the CP. Alternatively, Reelin may affect neurons at the end of migration and may provide a “detach and stop signal”, helping migrating neurons to disengage their interactions with the radial glia, which would then be free to guide the migration of the next wave of neurons [36,42,43]. Indeed, neurons deficient for the Reelin pathway remain attached to the radial glia during the entire course of locomotion, whereas wild-type cells detach from the glia fiber during the late stage of migration [42,44]. In addition, Reelin induces detachment and arrest of neurons migrating on radial glia fibers in vitro [43]. Finally, early born migrating neurons aberrantly invade the normally sparsely populated MZ in the reeler mouse, suggesting an abnormal over-migration [45]. Failure to detach from the radial glia would explain the “obstructed radial migration” observed in early studies [42], preventing the next waves of neurons from migrating past them. Further understanding on how neurons respond to Reelin and the discovery of the glia-independent somal translocation mode of migration suggested an adjustment of the “detach and stop signal”, replaced by the “detach and go model” [25,46,47,48,49]. In this model, Reelin still triggers the detachment from the radial glia but, in addition, induces a switch from the locomotion mode of migration into the terminal somal translocation mode when neurons get close to the MZ.

Some data seem to argue against these models. Ectopic expression of Reelin in the VZ of transgenic mice in the presence of endogenous Reelin did not alter neuronal migration or disorient the cells [50]. The same ectopic expression in reeler mice was able to rescue the splitting of the PP but not the layering of the CP. Similarly, the addition of Reelin to a reeler brain slice in culture partially rescues the reeler phenotype, with induction of PP splitting and development of a better-defined CP [51]. These data argue against a function of Reelin as a positional attractant cue or as a stop signal or even as a signal that triggers a switch in migration type since Reelin did not induce an ectopic terminal somal translocation before neurons reach the top of the CP. Several explanations could be suggested: (1) Reelin may act cooperatively with another signal that is localized at the top of the CP; (2) Reelin may be a permissive signal allowing neurons to respond to another signal localized near the MZ; (3) vice versa, another cue may be necessary for enabling neurons to respond to Reelin only when reaching a specific location in the cortex; or (4) a defined maturation or differentiation level of neurons might need to be acquired during the process of migration for them to respond in a specific manner to the Reelin stimulus [36]. Those are only speculations and still need to be assessed.

As already mentioned, abnormalities in the reeler cerebral cortex are more complicated than a simple inverted pattern of lamination [8,39]. Therefore, these models might only partly explain the effects of Reelin on migrating neurons. Maybe we were missing a piece of the puzzle. These models are focused on a function of Reelin on neurons near the top of the CP where it is highly concentrated. However, earlier studies indicate that, while the full-length Reelin is produced and mainly located at the MZ, cleavage fragments of Reelin and most importantly its active central fragment diffuse from the MZ into the depth of the tissue [51,52] and are able to reach cells in the multipolar morphology zone. Multipolar neurons express the intracellular adaptor Disabled 1 (Dab1) and the highest level of functional Reelin receptors [53,54,55]. These data suggest that Reelin may act before the start of the locomotion mode of migration. It was later found that Reelin affects neurons at the multipolar migration stage [56,57]. The “polarity” model was then suggested as an addition to the “detach and go” model [58]. During the multipolar mode of migration, neurons exhibit several neurites that elongate and retract actively. They switch the direction of movement in radial (both apically and basally) and tangential directions, but their net movement is still oriented towards the CP [30,31,56,59]. In vivo experiments, time lapse videomicroscopy in organotypic and lattice cultures and measurements of Golgi orientation demonstrated that the inhibition of intracellular signals induced by Reelin reduces the movement of multipolar neurons towards the CP and increases the tangential movements whereas the speed of migration is not affected [56,57]. The switch from multipolar glia-independent to bipolar glia-dependent locomotion was also delayed, resulting in an overall delay in the migration into the CP. In the absence of the polarizing cue and the signal to switch the mode of migration provided by Reelin, neurons would fail to orient their movement towards the CP and would enter the CP in a disorderly manner, disregarding their date of birth. On the other hand, once neurons are able to become bipolar and succeed to enter the CP, locomotion is not affected by inhibition of the Reelin pathway, as also observed in other studies [47,48,60]. The cell positioning phenotype in the reeler cerebral cortex is therefore the sum of at least two effects of Reelin during multipolar migration and terminal somal translocation.

### 2.3. Cell Mispositioning and Mechanism of Action of Reelin in the Hippocampus

The hippocampal formation is part of the cerebral cortex and comprises the hippocampus proper (the Ammon’s horn and the dentate gyrus), entorhinal cortex, parasubiculum, presubiculum and subicular complex. It is functionally associated with spatial learning as well as short- and long-term memory. The Ammon’s horn, or cornu ammonis (CA), is divided into the CA1, CA2 and CA3 regions, while the dentate gyrus contains the fascia dentata and the hilus.

Pyramidal neurons in the hippocampal CA1 region are mostly generated from E12 to E18 in the murine Ammonic VZ. They transform into multipolar cells in the intermediate zone, moving towards the hippocampal plate. When reaching the hippocampal plate, they switch to a bipolar migration along radial glia fibers, climbing from one fiber to another in a zigzag manner [38,61]. Pyramidal neurons in Ammon’s horn are thought to be roughly arranged in a birth-date-dependent inside-out manner [62]. In the reeler hippocampus, this layered organization is disrupted and two pyramidal layers are present instead of one [8,63].

In mice, few dentate granule cells are produced at E10 while the peak of neurogenesis is from E16 to the first postnatal week [64]. Newly generated dentate granule cells migrate along radial glial fibers from the primary dentate VZ toward the dentate anlage or, later, from the second proliferation zone to the dentate gyrus [65,66,67]. In contrast to the migration in the neocortex, dentate granule cells do not form layers in an inside-out fashion, but rather late-generated granule cells settle beneath the earlier-formed granule cells [63]. In the reeler mouse, the dentate gyrus is less densely packed and the number of granule cells is reduced [8,63,64]. Reelin is expressed at the hippocampal MZ by Cajal–Retzius cells. In the absence of Reelin signaling, a regular radial glia scaffold does not form while dentate granule cells fail to migrate into a compact cell layer but rather distribute all over the dentate hilar region [8,68]. In a hippocampal slice co-culture assay, Reelin can rescue radial glia fiber orientation and granule cell lamination only if correctly placed near the MZ [69,70]. Dentate granule cells in the reeler hippocampal slice migrate towards the source of Reelin and form a densely packed cell layer close to Reelin, which function here as an attractive cue. This is in contrast with the ability of Reelin to rescue CP development in the neocortex when simply added to the culture medium of organotypic brain slices without any directionality [51], suggesting differences in the mechanisms of action.

### 2.4. Cell Mispositioning and Mechanism of Action of Reelin in the Cerebellum

The cerebellum of reeler mice exhibits a neuronal disorganization and is severely hypoplastic. Because the cerebellum plays an important part in the control of motor coordination and precision, its malformation in the reeler mouse and in humans with REELIN mutations is considered the main cause of gait, ataxia and hypotonia [1,71,72]. However, some motor skill defects are independent of the cerebellum. A recent study found that conditional inhibition of the Reelin pathway specifically in the cerebral cortex but without affecting the cerebellum induces cortex-dependent, cerebellum-independent motor impairments that also seem independent of peripheral motor neural functions [73].

Faulty Reelin signaling in the cerebellum results in a defective migration of Purkinje cells and secondarily leads to a reduced proliferation of granule cell precursors. Purkinje cells are produced at the cerebellar VZ from E10.5 to E12.5 in mice and from GW 7 to GW 13 in humans [74,75]. Most of them migrate from the VZ to the cerebellar mantle along radial glia fibers (the precursors of Bergmann glial cells) to form the Purkinje cell plate, while some early born Purkinje cells are able to migrate independently of radial glia [76,77,78]. From there, they can stimulate the proliferation of granule cells, located just superficial to them in the external granule layer (EGL), mostly through the production of Sonic hedgehog (Shh) [79]. Granule cells are born in the EGL from E17.5 until postnatal day 16 (P16) in mice and migrate radially into the internal granule layer (IGL) until P20 [74]. In humans, the EGL forms from GW 10 until the 4th postnatal month, followed by the formation of the IGL until the 11th postnatal month [80]. Reelin begins to be expressed near the pial surface on E13.5 in the mouse by prospective deep nuclear neurons and then by EGL cells [40,81,82]. While delamination of postmitotic Purkinje cells from the ventricular epithelium and their initial migration is independent of Reelin signaling, their radial glia-guided migration absolutely requires an intact pathway since, in the absence of Reelin, Purkinje cells are unable to ascent radially and to form the Purkinje cell plate [76,83]. From their abnormally deep position, Purkinje cells are incapable of stimulating granule cell precursor proliferation, resulting in a selective depletion of granule neurons and hypoplasia of the cerebellum [84,85]. Studies on early born Purkinje cells derived from the posterior lateral cerebellum suggest a model in which Reelin triggers a switch in migration orientation. This subset of Purkinje cells that initiates Purkinje plate formation migrates tangentially from the posterior to the anterior lateral cerebellum. They then switch into a radial posture necessary for their radial migration towards the pial side in a Reelin-dependent manner. It is not known whether Reelin affects similarly the late arriving Purkinje cells [78,84].

### 2.5. Cell Mispositioning and Mechanism of Action of Reelin in the Olfactory Bulb

The olfactory bulb consists of five individual layers: the glomerular layer, the external plexiform layer, the mitral cell layer, the internal plexiform layer and the granule cell layer. These layers are formed in an inside-out manner during development [86]. At the embryonic stage, interneurons have been shown to arise from the lateral ganglionic eminence and dorsal telencephalon, whereas postnatally, they derive from the anterior part of the subventricular zone of the lateral ventricle [87]. Neuroblasts migrate tangentially to the olfactory bulb along the so-called rostral migratory stream (RMS) using a chain-like migration that involves interactions between migrating cells and tube-like structures formed by specialized astrocytes [88]. In these chains, neuroblasts migrate along each other in a saltatory fashion. Once in the olfactory bulb, neuroblasts switch from chain migration to radial migration, integrate into the laminated structure of the bulb, and differentiate into granule and periglomerular interneurons [89]. In adult reeler mice, olfactory bulbs are smaller because of a reduction in the number of neurons and exhibit a disorganization of the granular cell layer [86].

Reelin is mainly expressed in mitral cells (the principal projection neurons) and in some periglomerular neurons [90]. The switch from chain migration to radial migration seems to depend on the Reelin signal that detaches neuroblasts from the chains [91,92]. Components of the Reelin signaling pathway, very low-density lipoprotein receptor (VLDLR), Apolipoprotein E receptor 2 (ApoER2) and Dab1, are also important for the chain formation in the RMS but independently of Reelin which is not present in the stream [93]. Instead, it is Thrombospondin-1 that binds to the Reelin receptors, induces Dab1 phosphorylation but not Dab1 degradation or PKB (Protein kinase B, also named Akt) phosphorylation and stabilizes neuronal chain migration [94].

## 3. Reelin Signaling and Neuronal Migration

### 3.1. Reelin Structure and Proteolytic Processing

**Figure 1:**
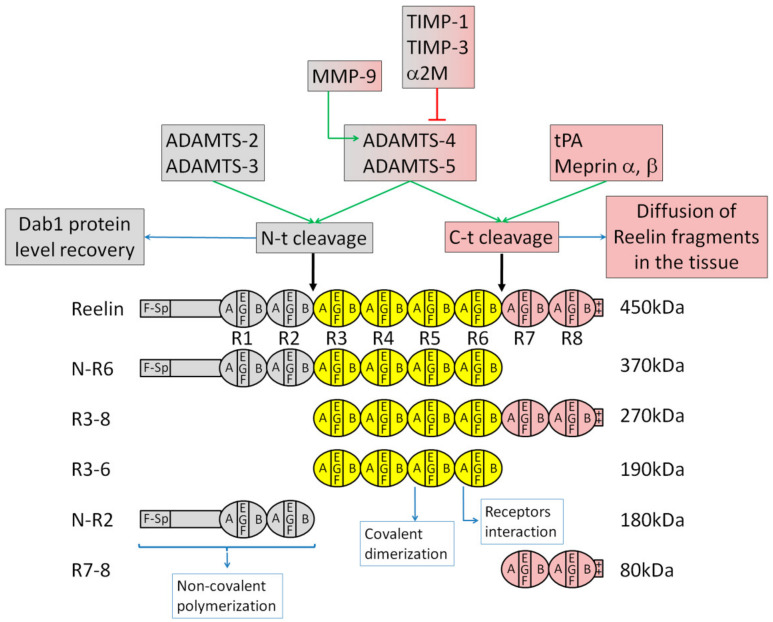
Structure of Reelin and its processing fragments: Reelin is a large extracellular matrix protein of 450 kDa. The protein starts with a signal peptide followed by an F-spondin homology domain (F-sp) and a unique region. The main body consists of eight Reelin-specific repeats: R1 to R8, each composed by two sub-repeats (A and B) flanking an EGF-like motif (EGF). Reelin ends with a basic stretch of 33 amino acids (++). After its secretion, Reelin is cleaved at two major sites to produce 5 fragments named N-R6, R3-8, R3-6, N-R2 and R7-8. R3-6 is the smallest biologically active fragment. Interaction with Apolipoprotein E receptor 2 (ApoER2) and very low-density lipoprotein receptor (VLDLR) occurs through the first subdomain of R6. Covalent homo-dimerization, which occurs through the first subdomain of R5, is not necessary for receptor interaction but is needed to induce Dab1 phosphorylation. N-R2 is involved in non-covalent polymerization. Proteases involved in the N-terminal (N-t) cleavage between Reelin repeat domains R2 and R3 are shown in grey. Proteases involved in the C-terminal (C-t) cleavage between Reelin repeat domains R6 and R7 are shown in red. Proteases involved in both cleavages are shown in both grey and red. A third cleavage by proprotein convertases removes six amino acids at the C-terminus of Reelin (not shown). The N-terminal cleavage does not prevent the early and midterm events of Reelin signaling but rather might be important to allow Dab1 protein level recovery, preventing an excessive long-term downregulation of the intracellular signal. C-terminal processing is important during the development of the neocortex to allow the active central fragment of Reelin (R3-6) and the N-R6 fragment to diffuse within the tissue, enabling them to reach and trigger the signal in multipolar neurons.

It was only in 1995 that the mutated gene of the reeler mouse was mapped and named *reelin* [40,95,96]. The reeler “Edinburg” strain has a complete loss of Reelin transcription, while the reeler “Orleans” strain expresses a Reelin protein that is not secreted due to a truncated C terminal end [95,97]. The *reelin* gene is conserved in many vertebrate species, including humans. Comparison between the mouse and humans shows that the amino acid and nucleotide sequences are 94.2% and 87.2% identical, respectively [98]. The Reelin protein is composed of 3461 amino acids with a deduced relative molecular mass of 388 kDa, while the glycosylated protein is around 450 kDa [40,52]. After a signal peptide followed by an F-spondin homology domain and a unique region, the main body consists of eight Reelin-specific repeats (R1 to R8, each composed by two sub-repeats flanking an epidermal growth factor (EGF)-like motif) and ends with a basic stretch of 33 amino acids (Figure 1) [40].

Early studies found that, after its secretion, Reelin is processed at two major sites between the second and third Reelin repeats (N terminal cleavage) and between the sixth and seventh repeats (C terminal cleavage), producing five fragments (named N-R2, R3-6, R7-8, N-R6 and R3-8) that could be observed using antibodies against N-terminal, central and C-terminal epitopes (Figure 1) [51,52,99]. The cleavage of Reelin can be found in developing [52] and adult brains [100,101]. Later works identified the cleavage sites more precisely [102,103]. Using specific inhibitors in vitro or in organotypic brain slice cultures, it was found that the processing depends on metalloproteinase activity secreted by cortical neurons [52,99]. In agreement with these studies, several potential candidates have been identified as enzymes that process Reelin (Figure 1). The extracellular matrix metalloproteinases ADAMTS-4 (a disintegrin and metalloproteinase with thrombospondin motifs-4) and ADAMTS-5 are able to cleave Reelin at both the N- and C-terminal sites while the serine protease tissue plasminogen activator (tPA) cleaves Reelin at its C-terminal site [100,104]. Processing of Reelin was abolished by the metalloproteinase inhibitors tissue inhibitor of metalloproteinases 1 (TIMP-1), TIMP-3 and α-2-macroglobulin. Matrix metalloproteinase 9 (MMP-9) also induces Reelin processing at both sites but indirectly through the activation of ADAMTS-4. Other studies found ADAMTS-2 and ADAMTS-3 as the enzymes that cleave Reelin at the N-terminal site and Meprin α and β as candidates responsible for the C-terminal cleavage [103,105,106]. Altogether, these data show that each processing site depends on the activity of several enzymes which could explain the absence of an effect on Reelin processing in tPA or Meprin β knockout mice [101,103].

The two processing sites carry different physiological significances. Early studies claimed that the processing of Reelin at the N-terminal site compromises Dab1 phosphorylation [107,108]. However, a more recent model suggests that the N-terminal cleavage regulates the duration of Reelin signaling. A mutated Reelin, uncleavable at its N-terminal site, and wild-type Reelin induce similar levels of Dab1 phosphorylation followed by a similar subsequent Dab1 degradation after several hours of stimulation in cultured neurons [102]. However, in a longer-term stimulation, up to 24 h, Dab1 downregulation is stronger with the mutated Reelin compared to the wild-type protein. In agreement, stimulation of neurons or of reeler brain slices by a Reelin deleted of its N-terminal fragment induces similar Dab1 phosphorylation and Dab1 degradation as full-length Reelin [51]. Therefore, N-terminal processing does not prevent the early and midterm events of Reelin signaling but rather might be important to allow Dab1 protein level recovery, preventing an excessive long-term downregulation of the intracellular signal. A reduced N-terminal processing seems to have only a mild effect in vivo [109]. A knock-in mouse expressing the mutated Reelin, uncleavable at its N-terminal site, exhibits a normal structure and layer organization in the cerebral cortex and cerebellum. On the other hand, dendrites are longer and more branched in cortical layer V neurons. The N-terminal processing might therefore be dispensable for neuronal migration while it plays a role in dendritic growth, at least in the cerebral cortex. Some hippocampal neurons are ectopically located in the knock-in mouse, reflecting differences with the cerebral cortex in mechanisms and in vulnerability to changes in Reelin proteolytic processing.

**Figure 2:**
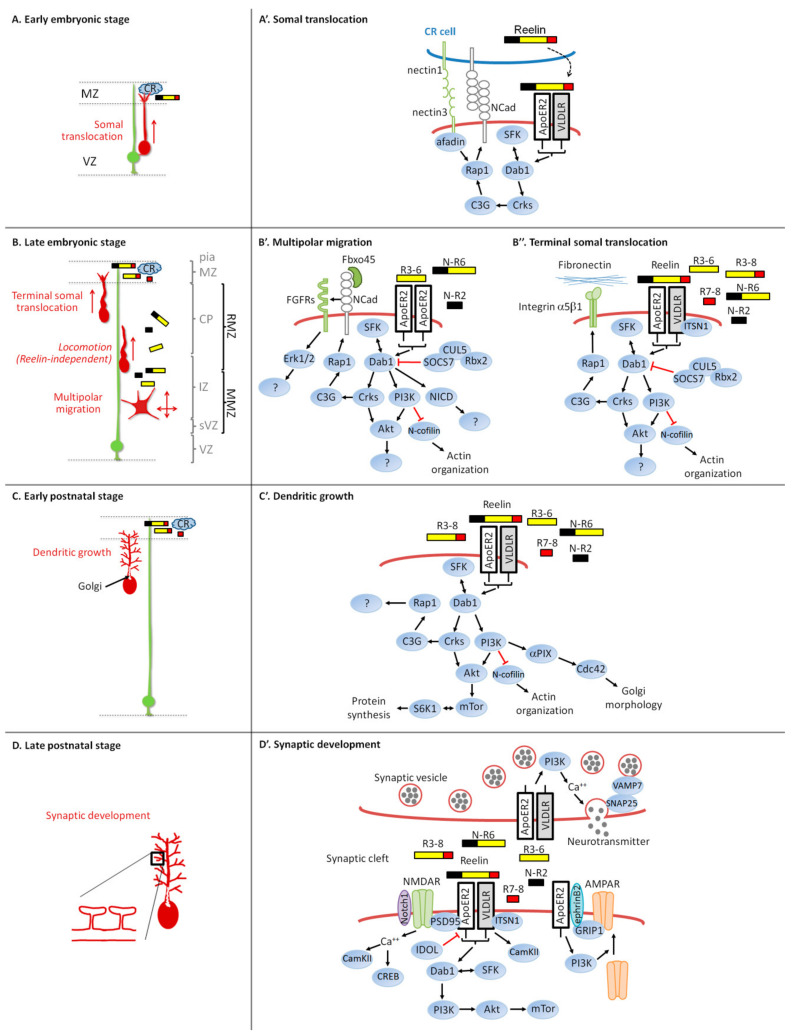
Modeling of the multiple functions of Reelin during migration, dendritic growth and synaptic development and the corresponding signaling pathways: (**A**,**A’**). During the early steps of cortical development, early born neurons perform a radial glia-independent somal translocation. Reelin triggers an interaction between Cajal–Retzius cells (CR) and the neuron leading process through N-cadherin and nectins. (**B**,**B’**,**B’’**). As the neocortex develops, the cerebral wall increases in thickness. The somal translocation mode of migration is gradually replaced by a radial migration subdivided into four steps: a short bipolar migration during which postmitotic cells move away from the ventricular zone (VZ) (not depicted here), followed by a radial glia-independent multipolar migration. Cells then switch to a radial glia-dependent but Reelin-independent bipolar locomotion and finish their journey with a terminal somal translocation. While the full-length Reelin is produced and mainly located at the marginal zone (MZ), cleavage fragments of Reelin depleted of the C-t fragment, namely R3-6, N-R2 and N-R6, diffuse from the MZ into the depth of the tissue to reach and signal to multipolar neurons. R3-6 and/or N-R6 interaction with ApoER2 receptors induces the movement of multipolar neurons towards the cortical plate (CP) and a timely subsequent switch from multipolar to bipolar migration. This depends on a Reelin-triggered interaction and activation of N-cadherin with fibroblast growth factors (FGF) receptors (FGFRs) and the downstream effectors Extracellular signal-regulated protein kinases 1 and 2 (Erk1/2). When bipolar locomoting neurons reach the top of the cortical plate (CP), Reelin triggers the detachment from the radial glia and induces a switch from the locomotion mode of migration into the terminal somal translocation. This requires the interaction of full-length Reelin and/or any of its processing fragments containing the central part R3-6 with VLDLR and ApoER2 to stimulate the interaction of Integrins α5β1 on the neuron leading process with fibronectin located at the MZ. Both multipolar migration and terminal somal translocation require activation of the small GTPase Rap1 and of the Phosphoinositide 3-kinase (PI3K)/Akt/n-cofilin pathway. (**C**,**C’**). After completion of migration, the leading process transforms into apical dendrites. Reelin triggers dendritic growth and branching in the cerebral cortex and hippocampus. Several mechanisms are involved. All these events seem to be regulated by PI3K: Activation of the mammalian target of rapamycin (mTor)/S6 kinase 1 (S6K1) pathway that could regulate protein synthesis; regulation of the actin cytoskeleton through n-cofilin; and promotion of the translocation of the Golgi into the longest dendrite in a Cdc42-dependent manner. (**D**,**D’**). Dendritic spines are small protrusions arising from the dendritic shaft where most excitatory synapses reside. Reelin regulates their density in the hippocampus and the cerebral cortex. Synaptic plasticity is the ability of a synapse to modify its strength and is also influenced by Reelin that accumulates at synaptic contacts. N-methyl-D-aspartate (NMDA) and α-amino-3-hydroxy-5-methyl-4-isoxazolepropionic acid (AMPA)-type glutamate receptors (NMDARs and AMPARs ) are two postsynaptic ionotropic receptors directly involved in synaptic plasticity. Reelin modulates the subunit composition of NMDARs, its phosphorylation, its calcium conductance and potentiation. Reelin also facilitates the insertion of AMPARs in postsynaptic membranes and presynaptic release of neurotransmitters. Core components VLDLR and/or ApoER2, Src family kinases (SFK) and Dab1 are involved in most Reelin functions. Dab1 is polyubiquitinated by an E3 ubiquitin ligase complex containing Cullin 5 (Cul5), Rbx2 and SOCS7 and is subsequently degraded by the proteasome. This rapid downregulation of the signal is necessary for a correct organization of the CP. Its function in Reelin-induced dendritic growth and at the synapse has not been investigated. The PI3K/Akt pathway is also involved in all these Reelin functions but was apparently not crucial for early somal translocation. See the text for more details.

In another model, C-terminal processing might be important to allow the active central fragment of Reelin and N-R6 to diffuse within the tissue, enabling them to reach and trigger the signal in multipolar neurons (Figure 2B). Indeed, the central fragment, containing Reelin repeats three to six, is necessary and sufficient to perform Reelin’s functions [51,52]. It is as efficient as full-length Reelin in binding to its two receptors VLDLR and ApoER2, in triggering Dab1 phosphorylation and in inducing the formation of a well-organized CP in a Reelin-deficient organotypic brain slice. These data suggest that Reelin processing does not inhibit its function. Interestingly, in mutant mice that do not express any of the two receptors for Reelin, an increase of the central fragment at the protein level is observed while the other fragments and full-length Reelin protein levels do not change [52]. This suggests that, in the wild-type brain, R3-6 might be internalized and degraded after binding to its receptors. Indeed, Reelin [110] and the central fragment [52] were shown to be internalized through VLDLR and ApoER2. In addition, inhibition of Reelin processing in organotypic brain slices prevents the phosphorylation of the intracellular adaptor Dab1 and induces a defect in the formation of the CP resembling the defect seen in a reeler cortical slice [52]. These data suggest that Reelin processing is important for the formation of the CP. Interestingly, inhibition of both N-t and C-t cleavages has no effect on Reelin-induced Dab1 phosphorylation in cultured dissociated cortical neurons. This difference could be explained by the following model: In cultured dissociated neurons, Reelin does not need to be processed to reach and stimulate neurons. However, in the organized tissue, Reelin might be hooked to the extracellular matrix at the top of the CP, and the processing might be important in order to free the central active fragment and to allow its diffusion to reach the target cells. Indeed, immunofluorescent staining using antibodies against different Reelin epitopes revealed that processing fragments containing the N-terminal and/or central epitopes (N-R6, R3-6 and/or N-R6) diffuse from the site of Reelin secretion at the MZ into the CP and the intermediate zone of the embryonic cerebral cortex, while fragments containing the C-terminal epitope (therefore including the full-length Reelin, R3-8 and R7-8) are only localized at the MZ (Figure 2B) [52]. Later, another study corroborated these results. Using an antibody that recognizes unprocessed Reelin, they show that the full-length protein is only present near the Cajal–Retzius cells while an antibody against an N-terminal epitope shows a staining deeper in the tissue [102].

An attractive possibility is that the combination of full-length Reelin and its processing fragments, located at the top of the CP, trigger an intracellular signal different from the signal induced by the specific processing fragments present at the intermediate zone. A recent work using a heterologous overexpression system in 293T cells suggests that full-length Reelin or its central processing fragment could trigger different signals due to differences in homo- versus hetero-oligomerization of the two receptors [111]. Changes in the expression of Reelin receptors might therefore cause differences in the signal since multipolar neurons mainly express ApoER2 and terminally translocating bipolar neurons express both VLDLR and ApoER2 [55]. Further investigation is required to understand these additional causes of signaling diversity.

The physiological importance of Reelin processing was also found in the hippocampus. Widening of the granule cell layer in the dentate gyrus is a pathological dispersion of granule cells and is frequently observed in patients with mesial temporal lobe epilepsy. Proteolytic processing of Reelin is required for the maintenance of granule cell lamination in the dentate gyrus [112,113]. In addition, epileptic conditions inhibit matrix metalloproteinase activity by upregulation of endogenous TIMP-1, which in turn leads to extracellular accumulation of uncleaved Reelin [114]. Finally, widening of the granule cell layer is accompanied by a loss of Reelin-expressing cells in the hippocampus of patients with mesial temporal lobe epilepsy [115], and the Reelin central processing fragment is able to prevent granule cell dispersion induced by kainite injection, an established model for mesial temporal lobe epilepsy [116]. Changes in the levels of Reelin fragments are observed in both neuropsychiatric and neurodegenerative disorders. An increased processing of Reelin was found in patients suffering from frontotemporal dementia, Alzheimer’s disease or Down syndrome [117,118]. On the other hand, it is decreased in blood samples of patients with schizophrenia, depression and bipolar disorder [117,119,120].

A third cleavage by proprotein convertases removes six amino acids at the C-terminus of Reelin [121]. It was only recently discovered because the resulting difference in molecular weight is too small to be discerned with SDS-PAGE. This processing is not required for neuronal migration in the neocortex during embryonic stages but influences the maintenance of the MZ in the postnatal cerebral cortex. In the absence of these six Reelin C-terminus amino acids, cerebellar formation is mostly normal with some Purkinje cells ectopically located [122].

Overall, these in vitro and in vivo data in mice and humans suggest a functional importance rather than an inhibitory function of Reelin proteolytic processing.

### 3.2. Intracellular Signaling Pathway and Neuronal Migration

Most Reelin functions described in this review, including neuronal migration, depend on the core Reelin signaling pathway (sometimes referred to as a canonical pathway) which includes Reelin receptors VLDLR and ApoER2, the intracellular adaptor Dab1, and the two Src family tyrosine kinases (SFK) Src and Fyn. Other downstream effectors are, on the other hand, more specific to defined tasks as summarized in Figure 2.

Reelin binds to the extracellular domain of the lipoprotein receptors VLDLR and ApoER2 [123,124]. For a recent review on the structure of these two receptors, please read Dlugosz et al. [125]. Several studies found that ApoER2 has a stronger affinity for Reelin when compared to VLDLR [126,127,128], while others suggested a similar affinity for both receptors [124]. The interaction involves the central processing fragment R3-6 through two lysine residues on the first subdomain of the sixth Reelin repeat (Figure 1) [51,129]. In both VLDLR and ApoER2, the first low density lipoprotein receptor (LDLR) class A module is sufficient to bind Reelin. This interaction is thought to induce di- or multimerization of the receptors to trigger intracellular signaling such as the phosphorylation of Dab1 by Src and Fyn [51,130]. Receptor clustering using a bivalent agent that binds both VLDLR and ApoER2 in primary neurons [130] or by incubating reeler brain slices with anti-receptor antibodies [51] triggers Dab1 phosphorylation. However, the Dab1 phosphorylation induced by receptor dimerization is not sufficient to correct the reeler phenotype in brain slice cultures as Reelin does [51]. This supports either the potential involvement of an additional signal such as a co-receptor or the need to induce a bigger multimerization complex. Reelin multimerization might therefore be a way to increase the signal. Reelin non-covalent dimerization occurs through the N-terminal fragment, and it was postulated as the primary oligomerization domain [107]. This may seem to contradict with the ability of the central fragment of Reelin to bind to the two receptors, to induce Dab1 phosphorylation and to trigger an organized CP in reeler brain slices as efficiently as the full-length protein [51]. However, it is now known that Reelin covalently homo-dimerizes via its central region (Figure 1) [127]. While Reelin homo-dimerization is not needed to bind to its receptors, it is important for triggering Dab1 phosphorylation in cultured neurons.

Mice with double inactivation of the lipoprotein receptor genes coding for VLDLR and ApoER2 have a reeler-like phenotype, whereas single receptor gene mutations generate subtle but distinguishable migration phenotypes [45]. Despite the obvious overlap in function, the redundancy is only partial. Analysis of single mutants shows that the absence of VLDLR has most of its effects in the cerebellum whereas deficiency of ApoER2 predominantly affects the cortex and hippocampus. The authors claimed that none of the single mutant mice exhibit neuronal invasion of the MZ. However, two more recent papers found that VLDLR-/- as well as ApoER2-/- neurons over-migrate into the MZ [131,132]. Within the neocortex, their differential involvement in neuronal migration could be due to their expression patterns. VLDLR is selectively expressed in the distal portion of leading processes of neurons approaching the MZ at the end of their migration, whereas ApoER2 is expressed throughout the developing neocortex with the exception of the VZ but mainly localized to neuronal processes and cell membranes of multipolar neurons [45,55]. These data suggest that VLDLR is more important for the terminal somal translocation while ApoER2 is mostly involved in the effect of Reelin on multipolar cells. Other differences exist between the two receptors that might suggest dissimilarities in the downstream signal induced by the pathway. ApoER2 associates with lipid rafts and Reelin binding induces ApoER2 degradation via lysosomes, whereas VLDLR resides in non-raft domains of the cell membrane and is not degraded by Reelin interaction [110]. However, ApoER2 exhibits a slower endocytosis rate after binding to Reelin when compared to VLDLR. In addition, Reelin binding to ApoER2 induces α-secretase cleavage of the receptor, producing extracellular soluble fragments that could act as dominant negative receptors for Reelin. The authors found that VLDLR induces lysosomal degradation of internalized Reelin while ApoER2 does not. The cytoplasmic domain of ApoER2 but not of VLDLR comprises a proline-rich region of 59 amino acids coded by exon 19 in the mouse and that interacts with c-Jun N-terminal kinase (JNK)-interacting protein 1 (JIP-1), JIP-2, postsynaptic density protein 95 (PSD-95) and X11α [133,134]. However, exon 19 is dispensable for Reelin-induced Dab1 and Akt phosphorylation and for neuronal positioning, whereas it is important for the enhancement of long-term potentiation (LTP) in adult brains [135].

Reelin binding to its receptors induces the phosphorylation of Dab1 [136]. The PI/PTB (protein interaction/phosphotyrosine binding) domain of Dab1 interacts with The NPxY (Asp-Pro-any amino acid-Tyr) sequences in the cytoplasmic tail of VLDLR and ApoER2 and to phosphatidylinositol-4,5-bisphosphate (PI4,5P2) [45,137,138]. Mice with spontaneous or induced mutations in the Dab1 gene exhibit a reeler-like phenotype [139,140,141]. There is no additional cortical defect in mice that lack both Reelin and Dab1, suggesting that the two proteins function in a linear pathway [136]. Dab1 is phosphorylated on five tyrosine residues by Src and Fyn [142,143]. The two kinases can interact with tyrosine phosphorylated Dab1 through their SH2 domain and their activation by Reelin depends on the presence of Dab1 [143]. The clustering of Dab1 through VLDLR and ApoER2 therefore triggers a positive activation loop and a localized amplification of tyrosine kinase activity. Dab1 dimerization is sufficient on its own to induce phosphorylation [130,144]. Mice carrying mutations in Dab1 on the five important tyrosine residues, organotypic brain slices inhibited for SFK kinase activity using a specific small molecule inhibitor, and double knockout mice for Src and Fyn exhibit a reeler-like phenotype [142,145,146,147]. Alternative splicing of Dab1 regulates its phosphorylation and function during multipolar migration [148]. Following tyrosine phosphorylation, Dab1 is polyubiquitinated by an E3 ubiquitin ligase complex containing Cullin 5 (Cul5), Rbx2 and SOCS7 and is subsequently degraded by the proteasome [149,150,151,152]. This rapid downregulation of the signal is necessary for a correct organization of the CP. Ablation of Cul5, Rbx2 or SOCS7 in migrating neurons results in the accumulation of Dab1 protein and a cortical layering defect [151,152,153]. The overexpression of a stabilized mutant Dab1, resistant to Cul5-dependent degradation, causes a similar phenotype [154]. Defective neurons exhibit an over-migration although they do not enter the MZ [151,152,154]. A simultaneous mechanism that turns down the signaling pathway is the downregulation of Reelin receptors that occurs before neurons exit the multipolar migration zone [53,155]. Overall, these data show that multiple mechanisms are at play to downregulate the Reelin signal for a normal brain development.

The adaptor molecules Crk and CrkL physically interact with Dab1 phosphorylated at Tyr220 and Tyr232 [156]. Crk and CrkL double conditional mutant mice display the major anatomic features of reeler including, cerebellar hypofoliation, failure of Purkinje cell migration, absence of preplate splitting, impaired dendritic development, and disruption of layer formation in the hippocampus and cerebral cortex [157]. They are involved in the Reelin-induced activation and phosphorylation of C3G and Akt [156,157]. C3G is a guanine nucleotide exchange factor (GEF) that activates the small GTPase Rap1, which is also activated by Reelin in cultured neurons [156]. Rap1 is involved in different aspects of Reelin-dependent migration in the developing neocortex. In migrating cortical projection neurons, the small GTPase Rap1 regulates the orientation of multipolar migration towards the pial surface without affecting the speed of movement by maintaining N-cadherin on the neuron plasma membrane (Figure 2B,B’) [56]. The function of N-cadherin however does not depend on its homophilic adhesive properties but rather relies on its physical interaction with fibroblast growth factors (FGF) receptors (FGFRs, FGFR1 and FGFR2 being the most important), which prevents FGFRs from being ubiquitinated and degraded through the lysosome [57]. Inhibition of FGFRs affects the orientation of multipolar migrating neurons but has no effect on their morphology. How the N-cadherin/FGFR complex orients migration is still unknown but might involve the Reelin-induced FGFR-dependent prolonged activation of Extracellular signal-regulated protein kinases 1 and 2 (Erk1/2) [57]. The central processing fragment of Reelin was able to trigger this FGFR and Erk1/2 activation as efficiently as the full-length Reelin. The implication of Erk1/2 in the pathway is not clear. Some authors have found that Reelin stimulates Erk1/2 phosphorylation in cultured embryonic cortical neurons [57,158,159,160] while others have not [161,162,163]. Differences in protocols, timing of stimulation, concentration of Reelin or the background of mice could result in different levels of stimulation. The F-box protein Fbxo45 is secreted by an unconventional mechanism by cortical neurons and interacts with the extracellular domain of N-cadherin [164]. This interaction seems to be important for N-cadherin functions during multipolar migration, but the mechanism of action is still unclear. During the terminal somal translocation near the top of the CP, the intracellular Dab1-Crk/CrkL-C3G-Rap1 pathway is also involved, but this time, it regulates integrin receptors instead (Figure 2B,B’’) [165]. Here, the activation of Reelin signaling promotes neuronal adhesion to fibronectin localized in the MZ through Integrin α5β1. These data disagree with an older study that found a normal migration and layer formation when Integrinβ1 is removed specifically in migrating excitatory cortical neurons [166]. Finally, the glia-independent somal translocation of early born neurons is also regulated by Reelin-induced Rap1 activation and depends on an interaction between the leading process of translocating neurons and Cajal–Retzius cells (Figure 2A,A’) [167,168]. The authors suggest a model where Nectin1 on Cajal–Retzius cells and nectin3 on the leading process mediate an initial heterophilic interaction which is then stabilized by N-cadherin homophilic contacts induced by Reelin, facilitating the translocation of the nucleus. The adaptor protein Afadin, which binds to the cytoplasmic domains of all nectins, is also required for the stabilization of the leading process at the MZ.

Other critical intracellular events occur after Reelin binding to its receptors. The p85α subunit of PI3K (phosphoinositide 3-kinase) interacts with the intracellular adaptor Dab1 in a manner dependent on Reelin [169]. This leads to the activation of PI3K and the subsequent stimulation of Akt phosphorylation on the Thr308 residue by PDK1 (Phosphoinositide-dependent kinase-1) and on Ser473 by mTORC2 (mammalian target of rapamycin complex 2) [170,171]. PI3K/Akt activation is required for a correct development of the CP, for the Reelin-induced oriented migration of multipolar cortical neurons and for the regulation of the terminal somal translocation (Figure 2B,B’,B’’) [56,165,170]. Reelin-activated Akt induces the phosphorylation and activation of S6 kinase 1 (S6K1) by mTORC1 in an SFK- and PI3K-dependent manner. This has however no function during neuronal migration but is involved in Reelin-induced dendritic growth and branching (Figure 2C,C’) [170]. Activated Akt phosphorylates Glycogen synthase kinases beta (GSK3β) at Ser9, which is stimulated by Reelin in cultured neurons [171]. However, several studies found that regulation of GSK3β phosphorylation at Ser9 (or Ser21 for GSK3α) is not important for the control of radial migration and neurite development in the neocortex [172,173]. This suggests that alternative mechanisms for GSK3 inactivation might play a more prominent role than phosphorylation at these specific sites.

Another protein regulated by Reelin in a Dab1-, ApoER2- and PI3K-dependent manner is the actin-depolymerizing protein n-cofilin [174]. N-cofilin is inhibited and phosphorylated after Reelin stimulation, most likely by LIM kinase 1 (LIMK1). Phosphorylation of n-cofilin takes place in the leading processes of migrating neurons as they approach the Reelin-containing MZ. The authors suggest that this is important for Reelin to stabilize the leading process and to promote the terminal somal translocation (Figure 2B’). Later on, the same group found that a phospho-mimetic n-cofilin mutant partially allows reeler mutant neurons to exit the multipolar migration zone and to enter the CP [175]. N-cofilin could therefore also regulate the orientation of multipolar neurons under the control of Reelin (Figure 2B’’).

Intersectin-1 (ITSN1), a scaffold protein associated with Down syndrome, physically interacts with Dab1 and VLDLR but not ApoER2 [176]. ITSN1 collaborates with VLDLR to regulate pyramidal cell lamination and synaptic plasticity in the hippocampus. In the neocortex, double knockout of ITSN1 and ApoER2 results in invasion of the MZ, suggesting a potential function of ITSN1 in the regulation of terminal translocation (Figure 2B’’).

Notch intracellular domain (NICD) interacts with Dab1, and Reelin signaling inhibits NICD degradation via Dab1 [177]. As a consequence, Reelin-deficient mice have reduced levels of NICD. Using in utero electroporation, the authors found that, while electroporated neurons are arrested at the intermediate zone of reeler cortices, overexpression of NICD in these neurons partially rescues the migration defect and allows some electroporated cells to reach the upper CP (Figure 2B’). Notch1 signaling seems also to be important for the Reelin-induced normal development of the dentate gyrus radial glia and migration of granule cells [178].

A few other proteins have been shown to interact with Dab1 such as Lis1 (lissencephaly-1), Nckβ, NWASP (Neural Wiskott-Aldrich Syndrome Protein) and Dab2IP (Disabled homolog 2-interacting protein) and may potentially function in Reelin signaling [179,180,181,182].

## 4. Dendritic Growth Defect in the Reeler Mouse

The dendritic architecture is disrupted in both homozygous and heterozygous reeler mice even though neuronal positioning is normal in heterozygous reeler mice, demonstrating that the dendritic phenotype is not secondary to neuronal mispositioning [183,184,185,186]. In vitro, Reelin stimulates the generation of dendrites when added to hippocampal neurons [170,185,187]. Overexpression of Reelin in adult hippocampus accelerates granule neuron dendritic growth and maturation, while inactivation of the Reelin pathway decreases dendritic development [188]. In the developing neocortex, apical dendrites emerge by direct transformation of the leading process after completion of migration. Reelin signaling was shown to promote the outgrowth of apical dendrites of cortical neurons that reach the MZ [28,47]. Using time-lapse videomicroscopy on neocortical explants, a study found that reeler neurons abnormally retract their apical dendrites away from the MZ soon after migration arrest [189,190]. The addition of Reelin rescues their orientation and stimulates dendritic growth in the MZ. Apical dendrites are subdivided into compartments containing specific sets of proteins and enabling the differential processing of their respective synaptic inputs. Downregulation of Dab1 postnatally, after completion of development, results in a reduction in the enrichment of HCN1 and GIRK1 in the distal compartment of the apical dendrites of hippocampal CA1 and neocortical layer 5 pyramidal neurons [191]. However, this postnatal knockdown of Dab1 has no effect on dendritic morphology or the expression of proteins normally present in a uniform distribution throughout the apical dendritic arbor such as MAP2 and the GluR1 AMPA receptor subunit. Therefore, after its function in dendritic growth and branching, Reelin might also be required to distribute proteins specifically to the distal compartment.

## 5. Reelin Signaling and Dendritic Growth

Core components of the Reelin pathway, ApoER2, VLDLR, Dab1 and SFK, mediate the activity of Reelin on dendritic growth [47,170,185]. The implication of the Dab1 degradation pathway Rbx2/Cul5/SOCS7 has not been investigated in the regulation of dendritic growth. Downregulation of Crk family proteins also blocks dendritogenesis induced by Reelin in vivo and in cultured hippocampal neurons [157,187]. Inhibition of the downstream effector Rap1 in vivo leads to delayed arrival of cortical neurons at the top of the CP that exhibit a shorter apical dendritic tree [56].

A PI3K-Akt-mTor-S6K1 pathway acts downstream of Reelin to stimulate dendritic growth and branching in a Dab1 phosphorylation-dependent manner [170]. Here, activated mTOR phosphorylates its substrate S6K1, a kinase involved in the control of protein translation. In addition, Reelin triggers growth cone motility of major and minor processes of cultured neurons in a Dab1-, ApoER2-, PI3K- and Cdc42-dependent manner [192]. Those functions however do not depend on GSK3 activity [170,192].

Neuronal Golgi is polarized toward and into the longest and most complex dendrites. This positioning of the Golgi may be responsible for the increased growth of the longest dendrite because disruption of the Golgi structure equalizes the growth rate between different dendrites [193]. Reelin appears to regulate this Golgi polarization, since Reelin induces an extension of the Golgi apparatus into dendrites in cultured neurons and into the apical dendrites of hippocampal and neocortical pyramidal neurons in vivo [190,194]. This function is opposed by an LKB1-Stk25-GM130 signaling pathway that induces Golgi condensation. The Cdc42/Rac1 GEF α PIX (p21-activated protein kinase exchange factor alpha, also called Arhgef6) but not βPIX was found to be downstream of Reelin to promote Golgi translocation into dendrites of cultured hippocampal neurons [195]. CLASP2 (CLIP-associating protein 2) is a microtubule plus-end tracking protein enriched at the Golgi apparatus of cultured neurons and at the growth cones of their extending neurites [196]. The inhibition of CLASP2 decreases axon and dendritic length without affecting the morphology of the Golgi. CLASP2 mRNA level is upregulated in adult reeler mice and its knockdown inhibits neurite growth induced by Reelin on cultured neurons [197]. CLASP2 interacts with Dab1 but the phosphorylation state of Dab1 does not control CLASP2 binding, and it is not known whether Reelin influences this interaction. In vivo downregulation of CLASP2 in migrating cortical projection neurons leads to mislocalized cells in deep cortical layers. However, further investigation is needed to determine whether the function of CLASP2 in neuronal migration is regulated by Reelin.

## 6. Synaptic Plasticity Defect in the Reeler Mouse

After birth, Cajal–Retzius cells are progressively lost from the forebrain to almost complete disappearance at P14 in mice by undergoing selective cell death through apoptosis [198,199]. However, Reelin is still present and becomes predominantly produced at postnatal and adult ages by a subset of GABAergic interneurons in the cortex and hippocampus and by glutamatergic pyramidal neurons in layer II of the entorhinal cortex [120,200,201]. Reelin’s main role at these later ages is to regulate dendritic development, spine formation and synaptogenesis. Dendritic spines are small protrusions arising from the dendritic shaft where most excitatory synapses reside. Their density is reduced in hippocampal pyramidal neurons and in the frontoparietal and prefrontal cortices of heterozygous and homozygous reeler mice [202,203,204]. In adult-generated hippocampal granule cells, Reelin controls the shape, size and type of dendritic spines and the degree of the perisynaptic astroglial ensheathment that controls synaptic homeostasis [205].

## 7. Reelin Signaling and Synaptic Plasticity

The core components of the Reelin signaling pathway, VLDLR, ApoER2, SFK and Dab1, are also implicated in dendritic spine growth and maturation [202]. ApoER2’s roles in structure and function of synapses and dendritic spines are modulated by the cytoplasmic adaptor proteins X11α and PSD-95 [206]. Constitutive ApoER2 expression caused by deficiency of the E3 ubiquitin ligase IDOL (Increased Degradation of LDL Receptor Protein) is detrimental to proper dendritic spine morphogenesis and the plasticity of neural circuits [207]. Dab1 conditional knockout in adult mice results in a reduced size of dendritic spines [208]. Other molecules such as CamKII (Ca2+/calmodulin-dependent protein kinase II) might also be required to enable Reelin and VLDLR to alter dendritic spine density [209,210].

Synaptic plasticity is the ability of a synapse to modify its strength during learning and memory. Long-term synaptic plasticity is a generic term that applies to a long-lasting experience-dependent change in the efficacy of synaptic transmission. This can be observed experimentally in the form of long-term potentiation (LTP), which is an enhancement in signal transmission between two neurons when stimulated synchronously. The induction of LTP or of the opposite phenomenon, long-term depression (LTD), is associated with the enlargement or shrinkage of the spine, respectively. Reelin accumulates at synaptic contacts, and heterozygous reeler mice exhibit reduced LTP and impaired learning [211]. On the other hand, stimulation of hippocampal slices with Reelin enhances LTP [212], and mice overexpressing Reelin under the control of the CamKIIa promoter exhibit an increased LTP response, increased excitatory synaptic contacts and hypertrophy of dendritic spines, reflecting a higher level of synaptic activity [213]. Interestingly, when Reelin is knocked down postnatally, the learning deficit is not observed, suggesting that it is caused by a developmental phenotype rather than an acute loss of signaling in the adult brain [214]. However, in vivo intraventricular injection of Reelin increases synaptic plasticity and strengthen learning and memory [215]. Further investigation is needed to better elucidate the physiological mechanisms of Reelin at the synapse and its effect on memory formation.

N-methyl-D-aspartate (NMDA) and α-amino-3-hydroxy-5-methyl-4-isoxazolepropionic acid (AMPA)-type glutamate receptors (NMDARs and AMPARs) are two postsynaptic ionotropic receptors directly involved in synaptic plasticity of excitatory transmission [216,217]. Core components of the Reelin pathway are located in postsynaptic densities of excitatory synapses situated in the dendritic spine. Reelin mediates tyrosine phosphorylation of and potentiates calcium influx through NMDARs in primary cortical neurons in a Dab1- and SFK-dependent manner [218]. This Reelin-induced calcium influx increases phosphorylation and nuclear translocation of the transcription factor cAMP-response element binding protein (CREB). Dab1 knockdown in postnatal forebrain shows that it is required in vivo for dendritic spine size regulation, for synaptic plasticity and for associative learning [208]. ApoER2 forms a functional complex with glutamate receptors of the NMDA subtype and is required for Reelin-induced tyrosine phosphorylation of NMDA receptor subunits [135]. Genetic ablation of either ApoER2 or VLDLR leads to impaired memory function in mice [212]. Notch1 interacts with the ApoER2/NMDA receptor complex and may contribute in Reelin-mediated synaptic potentiation [219]. However, in the adult brain, Notch cleavage and the consequent production of NICD is not affected by Reelin signaling. NMDA receptors are heteromeric ligand-gated ion channels composed most commonly of NR1 and one of the four NR2 subunits (NR2A-NR2D) which confers different properties. Reelin treatment facilitates a developmental switch from NR2B to NR2A in hippocampal neurons during postnatal maturation in an SFK- and VLDLR/APoER2-dependent manner [220,221]. Levels of PSD-95, the NMDA receptor subunits NR2A and NR2B, and the phosphatase PTEN are decreased specifically in the postsynaptic density fraction obtained from heterozygous reeler mice [222]. The mTor pathway is involved in the ketamine-induced, NMDA-dependent rescue of synaptic plasticity during postnatal development of the prefrontal cortex of heterozygous reeler mice [203]. Overall, these data support a regulation of NMDAR functions by Reelin during synaptic development and plasticity.

Reelin application to adult mice hippocampal slices also leads to enhanced glutamatergic transmission mediated by AMPARs by increasing the AMPAR number in a PI3K-dependent but SFK-independent manner [223]. De novo insertion of AMPARs at the synapse depends on the multi-PDZ adaptor glutamate-receptor-interacting protein 1 (GRIP1) that binds ApoER2 in a complex with ephrinB2 and the GluR2 subunit of the AMPARs [224].

Finally, Reelin might modulate presynaptic release mechanisms. VLDLR and ApoER2 are present at the presynaptic surface, and acute Reelin application to hippocampal neurons enhances spontaneous neurotransmitter release [209,225]. The number of presynaptic vesicles is significantly increased in CA1 synapses of reeler mutants, while the number of presynaptic boutons is not changed [226]. SNAP25 and VAMP7, two proteins of the soluble N-ethylmaleimide-sensitive factor attachment protein receptor (SNARE) complex; ApoER2; VLDLR; PI3K; and an increase in calcium levels are involved in this control of transmitter release induced by Reelin, while SFK are dispensable [225,226].

## 8. Reelin and Human Neurological Diseases

Mutations in the human REELIN gene induce an autosomal recessive form of lissencephaly characterized by a disorganized neuronal layering, reminiscent to the defects observed in the reeler mouse, and an absence or reduction of convolutions along with cerebellar hypoplasia [71,72]. Affected patients show severe delay in cognitive development, hypotonia and epilepsy. Mutations in the human VLDLR gene cause similar but much milder features and are associated with the dysequilibrium syndrome [227,228]. No neurodevelopmental disorders related to ApoER2 mutations have been identified so far. Finally, a pentanucleotide ATTTC repeat insertion in the noncoding region of cerebellar-specific DAB1 transcripts causes spinocerebellar ataxia with cerebellar atrophy [229].

More subtle alterations in Reelin signaling have also been linked to the etiology of various neuropsychiatric disorders such as autism, schizophrenia, bipolar disorder, depression, mental retardation, Alzheimer’s disease and epilepsy. They may partially be the result of improper neuronal migration or impaired synaptic formation and plasticity, all regulated by Reelin.

The REELIN gene is consistently cited as gene hosting common variants associated with autism spectrum disorder [230]. Several genome-wide screens show the potential of REELIN as an important contributor to genetic risk in autism [231]. However, genetic data suggest that heterozygous polymorphisms in REELIN alone are insufficient to cause autism spectrum disorder and that secondary genetic or environmental factors are likely required for a diagnosis [232,233]. The levels of Reelin protein and mRNA are reduced approximately two-fold in brain extracts from patients with chronic psychosis like schizophrenia and autism, maybe due to hypermethylation of the promoter region [234,235,236,237,238,239]. Heterozygous reeler mice have 50% reduction in Reelin mRNA and protein levels and exhibit some behavioral disorders reminiscent of those seen in human psychosis [240,241]. Interestingly, these mice have reduced prepulse inhibition, which is an indication of disrupted sensorimotor gating. In contrast, mice with the Reelin gene knocked out only during adulthood do not show similar anxiety and sensorimotor deficits, suggesting a developmental origin of these behavioral phenotypes [214]. Temporal lobe epilepsy is often associated with widening of the hippocampal granule cell layer. A decrease in Reelin is associated with granule cell dispersion in mouse models for temporal lobe epilepsy [113,115]. In humans, heterozygous mutations in the REELIN gene that are associated with a decreased level of Reelin protein cause autosomal-dominant lateral temporal epilepsy [242].

Disruption of neuronal plasticity, eventually resulting in a net loss of synapses and leading to early memory and cognitive deficits, is implicated as an early pathological event in Alzheimer’s disease (AD) [243]. Changes in NMDARs and AMPARs in the initial stages of AD have been reported [244]. Therefore, the Reelin pathway may contribute to the pathophysiology of AD through its modulation of NMDARs and AMPARs. Accumulating evidence supports a link between AD and Reelin. Reelin signaling has been associated with synaptic dysfunction and the neuropathology of AD using transcriptomic and genomic approaches [245,246,247]. Reelin is depleted in the entorhinal cortex of human amyloid precursor protein transgenic mice and humans with AD [120]. The decrease in Reelin expression is also observed long before the onset of amyloid-beta (Aβ) pathology in the hippocampus of human Aβ precursor 695 transgenic mouse and during preclinical AD stages in the human frontal cortex [248].

Other intriguing links have been uncovered between AD and the Reelin pathway. Accumulation of intraneuronal tangles composed of hyperphosphorylated tau and the presence of extracellular plaques of amyloid peptide Aβ, a processing product of the amyloid precursor protein (APP), are two hallmarks of AD associated with progressive synaptic loss and neuronal cell death. Reeler mice have increased Tau phosphorylation, and the Reelin signal induces a reduction in Tau phosphorylation through an inhibition of GSK-3β [124,171,249]. Reelin decreases amyloidogenic APP processing in vitro and prevents Aβ-induced synaptic dysfunction [250,251]. In vivo, reduced Reelin expression in AD mutant mice crossed with heterozygous reeler mice results in enhanced amyloidogenic APP processing and precocious production of Aβ peptides, a significant increase in number and size of Aβ plaques as well as age-related aggravation of plaque pathology in double mutant compared with single AD mutant [252]. These mice also show increased accumulations of phosphorylated Tau-positive neurons around Aβ plaques in aging double mutant mice compared to AD mutant mice. The concomitant reduction in Reelin-mediated signaling might play a role in synaptic dysfunction associated with Aβ deposition. Consistently, an increased Reelin protein level in AD mutant mice crossed with Reelin-overexpressing mice delays amyloid deposits, reduces synaptic loss, improves LTP and rescues memory-associated cognitive impairment [253,254].

Dab1 physically interacts with the cytoplasmic domain of APP, and this interaction antagonizes Reelin signaling [255,256]. On the other hand, Reelin increases Dab1 interaction with APP, reducing Aβ production [250]. The interaction of the Reelin receptor ApoER2 with APP reduces APP endocytosis, favoring non-amyloidogenic APP processing [257]. In addition, VLDLR and ApoER2 polymorphisms have been associated with AD [258,259]. The ε4 allele of the apolipoprotein E (ApoE4) gene is the primary genetic risk factor for the late-onset form of AD [260]. ApoE4 impairs the recycling of ApoER2 at the synaptic surface, preventing Reelin signaling and its protective effect against Aβ-mediated impairment of synaptic plasticity [261].

## 9. Concluding Remarks

To summarize, Reelin regulates multiple steps involved in the development of the mammalian brain, from neuronal migration to dendritic growth, spine formation, synaptogenesis and synaptic plasticity. During migration of excitatory neurons in the neocortex, Reelin seems to only affect the glia-independent migration steps which include somal translocation of early born neurons and multipolar migration and terminal somal translocation of late born neurons. Several models have been proposed to explain the cellular and molecular mechanisms through which Reelin regulates radially migrating neurons in the cerebral cortex. These models are evolving, building up on each other in parallel with the still growing advances in our knowledge. It is now well established that the disorganization of the neocortex not only is due to an effect of Reelin on somal translocation but also results from a defect in multipolar neurons.

Core components of the pathway (VLDLR, ApoER2, Dab1 and SFK) are involved in most of Reelin’s functions but others diverge depending of the mechanism implicated. While Rap1 seems to be involved in the three types of migration influenced by Reelin and described in this review, the downstream effectors and mechanisms of action are different: N-cadherin and nectins regulate early somal translocation, N-cadherin and FGFRs control multipolar migration, and Integrins α5β1 support terminal somal translocation. Quite interestingly, N-cadherin and integrins are involved as adhesive receptors to stabilize the leading process and allow somal translocation near the MZ. On the other hand, N-cadherin adhesive properties are not implicated in its function during the orientation of multipolar migration towards the CP induced by Reelin. Instead, its interaction with and activation of FGFRs is critical. Stimulation of the downstream effector Erk1/2 seems important, but further investigation is needed to better understand the mechanism at play.

The PI3K/Akt pathway is another important downstream effector for Reelin-regulated neuronal migration, dendritic development and synaptic plasticity. Here, the downstream effector mTor appears to be dispensable for migration but is implicated in dendritic growth and at the synapse. During synaptic development, Reelin mostly regulates NMDARs and AMPARs at postsynaptic densities and the release of neurotransmitters at the presynaptic terminals.

Several levels of regulation exist to modulate the downstream signal induced by Reelin. Expression of the two Reelin receptors, VLDLR and ApoER2, differs in multipolar or terminally translocating neurons. These receptors induce common and distinct downstream signals due to divergence in interacting proteins or differences in homo- versus hetero-oligomerization. The signal is also regulated by the cleavage of Reelin. The N-terminal processing seems to be responsible for the recovery of the Dab1 intracellular adaptor in order to prevent an excessive downregulation of the intracellular signal. The C-terminal cleavage allows diffusion of the N-R6 and R3-6 fragments deeper in the tissue to reach and signal to multipolar neurons. The processing could also influence the multimerization of the protein into structures of different sizes, potentially triggering different types of signal.

In addition to its function on neuronal migration, Reelin directly promotes maturation of dendrites, dendritic spine formation synaptogenesis and synaptic plasticity. Dendritic development depends on the regulation of protein synthesis, organization of the actin cytoskeleton and the polarization of the Golgi apparatus opposed by other polarity signals. Finally, through the modulation of the formation, function and plasticity of synaptic circuits, Reelin directly influences learning and memory.

Studies in human brain from neuropsychiatric and neurodegenerative disorder patients suggest an importance of the Reelin signaling pathway and the processing of Reelin in the etiology of the diseases. Therefore, molecules involved in the pathway and in its regulation are potential targets for therapeutic intervention.

Understanding the intrinsic and extrinsic mechanisms that regulate neuronal migration and the formation, maintenance and plasticity of neuronal connectivity during normal development and in disease remains an important challenge in the field of developmental neuroscience. The Reelin pathway has been known for many years now, and I have no doubt that our understanding of its multiple functions during development and in the adult brain will continue to progress during the many years to come.
